# Effect of Serum Phosphate on the Prognosis of Septic Patients: A Retrospective Study Based on MIMIC-IV Database

**DOI:** 10.3389/fmed.2022.728887

**Published:** 2022-03-08

**Authors:** Zhaoyang Li, Tingwen Shen, Yi Han

**Affiliations:** ^1^Department of Intensive Care Medicine, The First Affiliated Hospital of Nanjing Medical University, Nanjing, China; ^2^The Health Management Center, The First Affiliated Hospital of Nanjing Medical University, Nanjing, China; ^3^Department of Geriatric Intensive Care Medicine, The First Affiliated Hospital of Nanjing Medical University, Nanjing, China

**Keywords:** sepsis-3, phosphate levels, prognosis, MIMIC-IV, septic shock

## Abstract

**Objective:**

To assess the effect of serum inorganic phosphate (Pi) on the prognosis of patients with sepsis.

**Methods:**

A retrospective analysis of patients with sepsis selected from the Medical Information Mart for Intensive Care (MIMIC)-IV database was performed. Sepsis was diagnosed according to the Third International Consensus Definition for sepsis and septic shock (Sepsis-3). The time-weighted values of the serum Pi measurements within the first 24 h of sepsis were analyzed. The association between serum Pi and in-hospital mortality was evaluated with a generalized linear model (log-binomial model).

**Results:**

The analysis of 11,658 patients from six intensive care units (ICUs) showed a nearly linear correlation between serum Pi and in-hospital mortality in all patients with sepsis, especially in those with acute kidney injury (AKI). The increase of serum Pi was related to a higher risk of AKI, higher norepinephrine doses, ICU mortality, and in-hospital mortality. The generalized linear model showed that serum Pi was an independent predictor for in-hospital mortality in all patients with sepsis even within the normal range. The adjusted risk ratios (RRs) were also significant in subgroup analyses according to kidney function, gender, respiratory infection, vasopressor use, and Sequential Organ Failure Assessment (SOFA) score.

**Conclusion:**

Higher levels of serum Pi, even within the normal range, were significantly associated with a higher risk of in-hospital mortality in patients with sepsis regardless of kidney function, gender, respiratory infection, vasopressor use, and SOFA score.

## Introduction

Sepsis is a complex condition that remains the major cause of morbidity and mortality worldwide, and the true global burdens of sepsis are likely much higher than reported (1, 2).

A better understanding of sepsis has been researched in the past three decades (3). In 2016, the Third International Consensus Definition for Sepsis and Septic Shock (Sepsis-3) defined sepsis as life-threatening organ dysfunction resulting from dysregulated host responses to infection, which offers greater consistency for epidemiologic studies and clinical trials (4). The hour-1-bundle proposed by Surviving Sepsis Campaign in 2018 encourages clinicians to act as quickly as possible to make an accurate diagnosis and start appropriate treatment if clinically indicated (5).

Although great progress has been made into the pathobiology, management, and epidemiology of the disease, the high mortality is still unacceptable (2). The early identification of patients with sepsis at high risk of death allows clinicians to administer treatment in time. Inorganic phosphate (Pi) plays crucial roles in several aspects of physiological processes including energy metabolism, cellular signal transduction, and membrane transport (6, 7). Serum Pi disorders are common in critically ill patients, which can be attributed to several factors including gastrointestinal dysfunction, acute kidney dysfunction, and redistribution of Pi from the extracellular space into the cells (8, 9).

The predictive value of serum Pi has been studied in several specific patient populations. It was reported that even a minor increase in serum Pi was associated with a higher risk of several adverse outcomes including worsened heart failure and all-cause mortality in patients with heart failure (10–12). Numerous studies have confirmed the association between higher serum Pi and adverse outcomes in patients with chronic kidney disease (CKD) with or without kidney transplantation (13, 14). In the general population, it was also reported that a minor increase in phosphate at the base level was associated with a significant increase in all-cause mortality (15, 16).

Though serum Pi was considered to be a good predictor of adverse outcomes for several diseases, its prognostic value for patients with sepsis has not been well investigated yet, and the conclusions are mixed. For instance, in Shor et al’s study, severe hypophosphatemia in sepsis increased the risk of death by nearly 8-fold (8). In contrast, in Miller et al.’s study, they concluded that patients with hyperphosphatemia had higher 28-day in-hospital mortality while those with hypophosphatemia did not (17).

Moreover, the heterogeneity of disease severity and classification are great in intensive care units (ICUs). Thus, it is of vital importance to re-evaluate the association between serum Pi and mortality in patients with sepsis and different septic subgroups. We aimed to assess the effect of serum Pi within the first day of sepsis on the prognosis of patients with sepsis. In particular, the subgroup analyses were performed according to the presence or absence of acute kidney injury (AKI) or CKD, gender, respiratory infection, vasopressor use, and Sequential Organ Failure Assessment (SOFA) score in this study.

## Materials and Methods

### Study Design and Participants

The Medical Information Mart for Intensive Care (MIMIC)-III database provided critical care data for over 40,000 patients admitted to ICUs at the Beth Israel Deaconess Medical Center (BIDMC) from 2001 to 2012. Importantly, patient identifiers were removed according to the Health Insurance Portability and Accountability Act (HIPAA) Safe Harbor provision. MIMIC-IV, an update to MIMIC-III, incorporates contemporary data and improves on numerous aspects of MIMIC-III. The project was approved by the Institutional Review Boards of BIDMC (Boston, MA, United States) and the Massachusetts Institute of Technology (Cambridge, MA, United States). The latest version of MIMIC-IV was available on the PhysioNet, an online forum for the dissemination and exchange of recorded biomedical signals and open-source software for analyzing them (Johnson, Alistair, et al. “MIMIC-IV” (version 1.0), PhysioNet (2021), https://doi.org/10.13026/s6n6-xd98) (18).

The database is accessible to researchers who have completed a ‘protecting human subjects’ training. The data presented in this study were extracted by author Li, who completed the online training course of Data or Specimens Only Research (certification number: 38455531). Data extraction was performed using PostgreSQL tools V 12.4.

Patients meeting the diagnostic criteria of Sepsis-3 and older than 18 years old were enrolled in the study. For clinical operationalization, organ dysfunction associated with sepsis can be represented by an increase [Sepsis-related] in the SOFA score of 2 points or more, which is associated with an in-hospital mortality greater than 10%, and patients with septic shock can be clinically identified by a vasopressor requirement to maintain a mean arterial pressure of 65 mmHg or greater and serum lactate level greater than 2 mmol/L (>18 mg/dl) in the absence of hypovolemia(4, 19).

### Data Collection

The following information was extracted: age, gender, weight, pre-ICU comorbidities [hypertension, diabetes, CKD, coronary artery disease (CAD)], AKI, hospital and ICU length of stay (HLOS and ILOS), in-hospital mortality, ICU mortality, SOFA score, vasopressor use, white blood cell count, Serum Pi, serum creatinine, and blood lactate, infection sites. The definition of vasopressor use was any use of the following vasopressors, including norepinephrine, epinephrine, dopamine, and dobutamine, within the first 24 h of sepsis. The diagnosis and staging of AKI were according to the criteria of the Kidney Disease: Improving Global Outcomes (KDIGO) AKI Guideline Work Group (20).

The measurements of serum Pi and blood lactate within 24 h after the diagnosis of sepsis were extracted, and the time-weighted mean (TWM) values were calculated to represent the serum Pi and blood lactate level over the course by taking the area under the time-value curve divided by the time between the first and the last measurement assuming a linear trend between measurements (17). The serum levels of creatinine and the white blood cell count at the diagnosis of sepsis were extracted. The normal range of serum Pi is 2.7–4.5 mg/dl in MIMIC IV. Thus, crude outcomes were compared among the three groups based on the TWM values of the serum Pi measurements: hypophosphatemia (<2.7 mg/dl), normophosphatemia (2.7–4.5 mg/dl), and hyperphosphatemia (>4.5mg/dl).

### Primary Exposure and the Primary Outcome

The primary exposure was serum Pi level and the primary endpoint was in-hospital mortality. The secondary endpoints included ICU mortality, development of AKI, HLOS, ILOS, SOFA scores at diagnosis of sepsis, and norepinephrine doses.

### Statistical Analysis

The data were analyzed using the software Stata V.12.1. Continuous variables are presented as mean ± SD or median with interquartile ranges (IQR) and compared by the Student’s *t*-test and the Wilcoxon rank-sum test as appropriate. Categorical variables are reported as numbers and percentages and the comparisons were analyzed by Chi-squared and Fisher’s exact tests. One-way ANOVA or the Kruskal–Wallis test was performed for comparisons of crude outcomes between the three groups. Locally weighted scatterplot smoothing (LOWESS) regression and logistic regression were used to explore the crude relationship between serum Pi and in-hospital mortality. It has been studied that the odd ratio always overstates the relative risk when the outcome incidence is common (>10%). A generalized linear model (log-binomial model) utilized to determine the adjusted RRs is statistically appropriate in this situation. This model is applied because the overall in-hospital mortality in this study was not rare (21.3%) (21).

The subgroup analyses were based on the diagnosis of CKD or AKI for the kidney is the main regulator of extracellular fluid Pi concentration by virtue of having a tubular maximum reabsorptive capacity for Pi (22). Also, subgroup analyses based on gender, respiratory infection, vasopressor use, and SOFA score were performed. In the subgroup analysis, according to SOFA score, the patients were divided into two groups according to the median of the SOFA score (3 in this study).

## Results

### Demographic Data and General Clinical Characteristics

**FIGURE 1:**
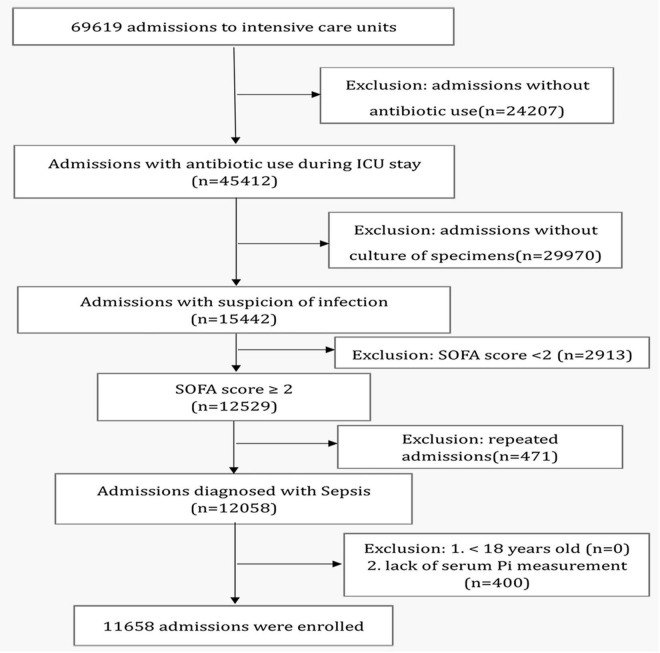
Study flowchart. ICU, intensive care unit.

The MIMIC-IV contains records for 524,520 admissions, of which 69,619 were admitted to ICUs. Of these, 54,177 admissions without suspicious infections were excluded for no antibiotic administration or culture of specimens during ICU stays. A total of 2,913 admissions were excluded for the SOFA scores were less than 2. A total of 12,529 admissions were considered to be patients with sepsis according to the definition of Sepsis-3. A total of 471 repeated admissions were excluded for we only take the patient’s first admission to ICUs. In total, 400 admissions were excluded due to the lack of serum Pi measurement. Finally, 11,658 admissions (9,179 survivors and 2,479 non-survivors) from 6 ICUs, including trauma surgical ICU, neurosurgical ICU, medical ICU, postanesthesia care unit, coronary care unit, and cardiac vascular ICU were analyzed. All enrolled patients were older than 18 years old (Figure 1).

The baseline characteristics of the survivors and non-survivors are presented in Table 1. The overall in-hospital mortality was 21.3%. The non-survivors were older (*p* < 0.01) and had lower weight (*p* = 0.01), higher SOFA scores (*p* < 0.01), higher white blood cell count (*p* < 0.01), higher serum creatinine levels (*p* < 0.01), higher blood lactate levels (*p* < 0.01), and higher serum Pi levels (*p* < 0.01). A higher percentage of patients had hypertension (*p* = 0.03), CKD (*p* < 0.01), CAD (*p* = 0.01), AKI (*p* < 0.01), and vasopressor use (*p* < 0.01) in the non-survivor group than in the survivor group. Respiratory infection seemed to be associated with higher in-hospital mortality compared with urinary system infection and blood system infection.

**TABLE 1:** Comparisons of demographics between survivors and non-survivors.

	Survivors (*n* = 9179)	Non-survivors (*n* = 2479)	*P* value
Age (years)	66.7 ± 16.2	70.2 ± 14.9	< 0.01
Female(*n*%)	4427(48.2%)	1169(47.2%)	0.34
Weight (kilograms)	81.8 ± 26.9	80.2 ± 24.7	0.01
SOFA score	3(2-4)	4(3-6)	< 0.01
Vasopressor use [*n* (%)]	2130(32.2%)	1132(45.7%)	< 0.01
Hypertension [*n* (%)]	1962(21.4%)	479(19.3%)	0.03
Diabetes [*n* (%)]	2470(26.9%)	637(25.7%)	0.23
CKD [*n* (%)]	2251(24.5%)	694(28.0%)	< 0.01
CAD [*n* (%)]	1387(15.1%)	429(17.3%)	0.01
WBC (*10^9^/l)	13.4 ± 10.9	15.3 ± 13.6	< 0.01
Creatinine (mg/dl)	1.77 ± 1.79	2.04 ± 1.66	< 0.01
Blood lactate (mmol/l)	1.92 ± 1.15	3.42 ± 3.06	< 0.01
Serum Pi (mg/dl)	3.96 ± 1.23	4.22 + 1.74	< 0.01
AKI [*n* (%)]	958(10.4%)	454(18.3%)	< 0.01
**Infection site [*n* (%)]**			
Respiratory system [*n* (%)]	2744(29.9%)	979(39.5%)	< 0.01
Digestive system [*n* (%)]	983(10.7%)	261(10.5%)	0.80
Urinary system [*n* (%)]	3057(33.3%)	621(25.1%)	< 0.01
Blood system [*n* (%)]	659(7.18%)	128(5.16%)	< 0.01
Other sites [*n* (%)]	3586(39.1%)	982(39.6%)	0.62

*BMI, body mass index; SOFA score, the Sequential Organ Failure Assessment; CKD, chronic kidney disease; CAD, coronary artery disease; WBC, white blood cell; Pi, inorganic phosphate; AKI, acute kidney disease.*

### Serum Pi and Outcome of Patients With or Without Kidney Dysfunction

**FIGURE 2:**
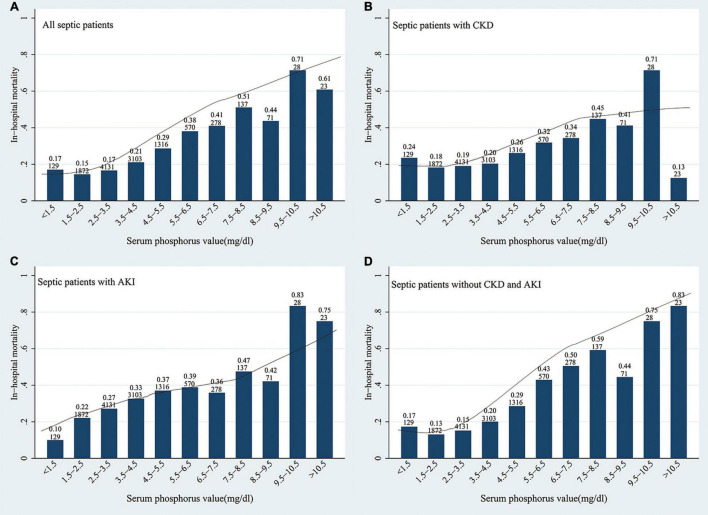
Association between serum inorganic phosphate (Pi) and the in-hospital mortality of patients with sepsis with **(A–C)** or without **(D)** chronic kidney disease (CKD) and acute kidney injury (AKI). A nearly linear relationship was found in this figure, especially in the overall population **(A)** and the AKI subgroup **(C)**. Pi, inorganic phosphate; CKD, chronic kidney disease; AKI, acute kidney injury. The legends on the top of each bar mean the in-hospital mortality and number of admissions in each serum Pi group.

The results for crude outcomes are listed in Table 2. These results revealed that hyperphosphatemia was associated with a longer duration of ICU stay (*p* < 0.01) and higher SOFA scores (*p* < 0.01). And the increase of serum Pi, even in the normal range, was related to higher risks of AKI (*p* = 0.01, *p* < 0.01), higher norepinephrine doses (*p* < 0.01, all), ICU death (*p* < 0.01, all), and hospital death (*p* < 0.01, all). Figure 2 shows the unadjusted relationship between serum Pi and in-hospital mortality using LOWESS smoothing technique in different kidney function subgroups. A nearly linear relationship was found in all patients with sepsis, especially those with AKI (Figures 2A,C). This relationship was less clear for patients with sepsis with CKD and those without AKI and CKD (Figures 2B,D).

**TABLE 2:** Unadjusted relationships between serum inorganic phosphate (Pi) groups and crude outcomes.

Serum Pi [mg/dl, (*n*)]	<2.7(2749)	2.7-4.5(6486)	> 4.5(2423)	P_1_P_2_ value
ICU stay [day, median (IQR)]	4.58(1.96–12.1)	4.57(2.0–11.8)	5.16(2.19-12.7)	0.42 <0.01
Hospital stay [day, median (IQR)]	10.7(6.09–19.4)	10.8(6.06–19.8)	11.7(5.98-21.8)	0.78 0.14
SOFA score [median (IQR)]	3(2–4)	3(2–4)	4(3-6)	0.11 <0.01
Norepinephrine rate [mcg/kg/min, median (IQR)]	0.12(0.06–0.24)	0.15(0.08–0.29)	0.25(0.10–0.42)	<0.01 <0.01
AKI [*n* (%)]	235(8.55%)	674(10.4%)	502(20.7%)	0.01 <0.01
ICU mortality [*n* (%)]	312(11.3%)	931(14.4%)	713(29.4%)	<0.01 <0.01
In-hospital mortality [*n* (%)]	415(15.1%)	1221(18.8%)	843(34.8%)	<0.01 <0.01

*P1 represents the p-value of comparisons between the hypophosphatemia group and the normophosphatemia group and p2 represents the p-value of comparisons between the normophosphatemia group and the hyperphosphatemia group; Pi, inorganic phosphate; SOFA score, the Sequential Organ Failure Assessment; AKI, acute kidney disease.*

### Serum Pi and In-Hospital Mortality of Patients With or Without Kidney Dysfunction

**FIGURE 3:**
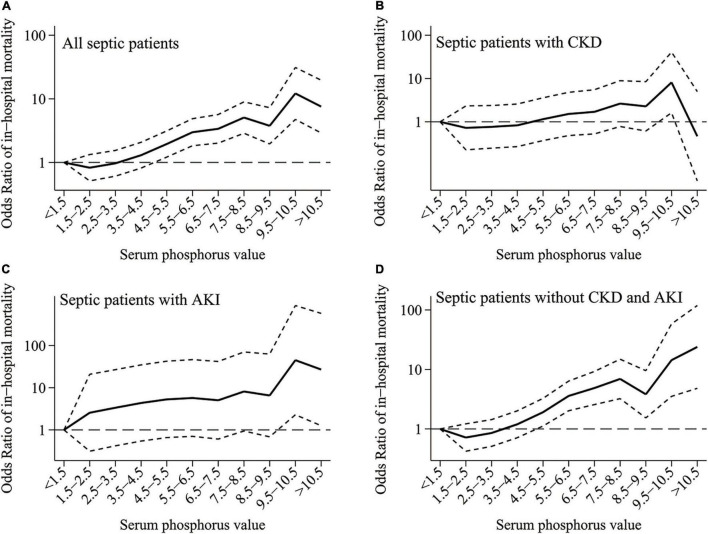
**(A–D)** Unadjusted odds ratios (Ors) of in-hospital mortality with <1.5 mg/dl as the reference group in patients with sepsis with CKD and AKI or not. The figure shows higher Pi was related to a higher risk of in-hospital mortality even in the normal range though it was only significant for extremely high value in the CKD and AKI subgroups. CKD, chronic kidney disease; AKI, acute kidney injury.

A logistic regression model with Pi < 1.5 mg/dl as the reference group was used to evaluate the unadjusted relationship between serum Pi and the risk of in-hospital mortality. The results were shown in Figure 3. It showed that the increase of Pi was related to higher odds ratios (ORs) of in-hospital mortality even in the normal range. But it was only significant for the extremely high values in the CKD and AKI subgroups (Figures 3B,C). Also, the OR of in-hospital mortality was higher among those with a serum Pi level less than 1.5 mg/dl compared with those with a serum Pi level between 1.5 and 2.5 mg/dl in the overall septic population and patients with sepsis without CKD and AKI though they are not statistically significant (Figures 3A,D).

### Predictive Value of Serum Pi for In-Hospital Mortality

**FIGURE 4:**
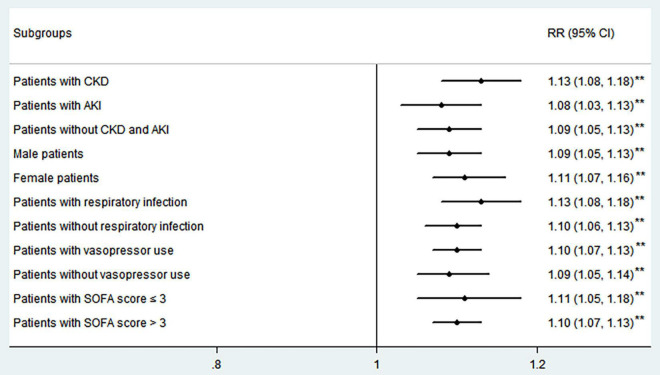
Adjusted risk ratio (RR) of serum Pi for in-hospital mortality in different septic subgroups. **p < 0.01; CKD, chronic kidney disease; AKI, acute kidney injury. SOFA score, sequential organ failure assessment score.

In order to eliminate the influences of possible confounding factors, an adjusted risk ratio was used to confirm the relationship between the Pi and in-hospital mortality. The results were shown in Table 3. Variables considered to be associated with in-hospital mortality and serum Pi homeostasis, i.e., variables with a *p*-value less than0.1 in Table 1 and gender in this study, were included in the analysis. After adjusting for other confounders, including age, gender, SOFA scores, weight, vasopressor use, hypertension, CAD, CKD, AKI, white blood cell (WBC) count, serum creatinine level, blood lactate level, respiratory infection, urinary infection, and bloodstream infection, the log-binomial model indicated that Pi was an independent predictor of in-hospital mortality (RR 1.11, 95%CI 1.08–1.23, *p* < 0.01). It means that a 1 mg/dl increase in Pi was associated with an incremental increase of 11% in in-hospital mortality. Older age, higher SOFA scores, lower weight, more vasopressor use, more AKI, lower serum creatinine levels, and respiratory infection were also significantly associated with an increased risk of in-hospital mortality. The same model was built for the subgroup analyses. The results showed that Pi performed well in these subgroups too (RR 1.13, 95%CI 1.08–1.18, *p* < 0.01 for patients with sepsis with CKD; RR 1.08, 95%CI 1.03–1.13, *p* < 0.01 for patients with sepsis with AKI; RR 1.09, 95%CI 1.05–1.13, *p* < 0.01 for patients with sepsis without CKD and AKI; RR 1.09, 95%CI 1.05–1.13, *p* < 0.01 for male patients; RR 1.11, 95%CI 1.07–1.16, *p* < 0.01 for female patients; RR 1.13, 95%CI 1.08–1.18, *p* < 0.01 for patients with respiratory infection; RR 1.10, 95%CI 1.06–1.13, *p* < 0.01 for patients without respiratory infection; RR 1.10, 95%CI 1.07–1.13, *p* < 0.01 for patients with vasopressor use; RR 1.09, 95%CI 1.05–1.14, *p* < 0.01 for patients without vasopressor use; RR 1.11, 95%CI 1.05–1.18, *p* < 0.01 for patients with SOFA score ≤ 3; RR 1.10 95%CI 1.07–1.13, *p* < 0.01 for patients with SOFA score >3. Figure 4).

**TABLE 3:** Results of log-binomial model analysis.

Variables	B	SE	Z	Adjusted RRs	*P* value
Age (years)	0.02	0.00	10.36	1.02(1.01–1.02)	< 0.01
Gender (Female)	–0.00	0.04	–0.07	1.00(0.92–1.08)	0.92
SOFA score	0.07	0.01	10.11	1.07(1.06–1.09)	< 0.01
Weight (kilograms)	–0.00	0.00	–2.38	1.00(1.00–1.00)	0.02
Vasopressor use	0.41	0.07	9.45	1.51(1.38–1.64)	< 0.01
Hypertension	–0.08	0.05	–1.54	0.92(0.83–1.02)	0.12
CAD	–0.04	0.05	–0.67	0.97(0.87–1.07)	0.50
CKD	–0.01	0.05	–0.17	0.99(0.90–1.10)	0.86
AKI	0.23	0.07	4.48	1.26(1.14–1.40)	< 0.01
WBC(*10^9^/l)	0.00	0.00	1.35	1.00(1.00–1.00)	0.18
Creatinine(mg/dl)	–0.05	0.01	–3.59	0.95(0.92–0.98)	< 0.01
Blood lactate(mmol/l)	0.09	0.01	13.16	1.10(1.08–1.11)	< 0.01
Respiratory infection	0.27	0.05	6.49	1.31(1.21–1.42)	< 0.01
Urinary infection	–0.27	0.03	–5.63	0.76(0.69–0.84)	< 0.01
Bloodstream infection	–0.18	0.07	–1.89	0.84(0.69–1.00)	0.06
Serum Pi(mg/dl)	0.10	0.01	7.68	1.11(1.08–1.23)	< 0.01

*SOFA score, sequential organ failure assessment score; BMI, body mass index; CAD, coronary artery disease; CKD, chronic kidney disease; AKI, acute kidney injury; WBC, white blood cell; Pi, inorganic phosphate.*

## Discussion

In this study, we found that serum Pi was an independent predictor of in-hospital mortality in the overall septic population and subgroups categorized according to kidney function, gender, respiratory infection, vasopressor use, and SOFA score. Even in the normal range, a minor increase of Pi was also associated with a higher risk of in-hospital mortality. However, this study did not reveal hypophosphatemia as a risk factor for mortality. To our best knowledge, this is the first research studying the relationship between serum Pi and in-hospital mortality in patients with sepsis with a large sample size.

The prognostic values of serum Pi were well studied in many diseases, while published studies in critically ill patients mainly focused on hypophosphatemia (8, 23–25). Most of the studies either included too few cases or were single-centered. The conclusions about the relationships between serum Pi abnormalities and ICU outcomes were inconsistent. In Shor et al.’s study, they reported that severe hypophosphatemia increased the risk of death by nearly 8-fold (8). However, only 55 patients were included. The algorithm for selecting and calculating the serum Pi levels was not clear. A recently published study including patients with bloodstream infection (BSI) from different ICUs concluded that hypophosphatemia was independently associated with a two-fold increase in 90-day mortality in ICU patients with BSI (24). However, the serum Pi level was assessed by only a single measurement obtained during ICU admission. Similarly, the conclusion of a large, multicenter retrospective study that hypophosphatemia at admission was independently associated with increased risk of death was questionable for hypophosphatemia was defined as at least one measurement meeting the criteria of hypophosphatemia and no measurement of hyperphosphatemia (26). In contrast, using a similar definition of hypo- and hyperphosphatemia, Broman et al. did not find any difference between the hypophosphatemic group and the control group (27). In our study, the TWM values of serum Pi were calculated. We think this algorism could reflect the level of serum Pi more accurately. Similar to another study by Suzuki et al. (28), we found that the OR of in-hospital mortality of severe hypophosphatemia (<1.5mg/dl in this study) was higher but not statistically significant in the overall septic population and patients with sepsis without CKD and AKI. Although conclusions regarding the effect of hypophosphatemia on the prognosis of critically ill patients were different, these studies agreed that hyperphosphatemia was an independent predictor of poor prognosis (26, 27). Several other studies also confirmed the association between hyperphosphatemia and adverse outcomes in critically ill patients (17, 29, 30). In these studies, the higher risk of hyperphosphatemia for unfavorable outcomes was all compared to the normophosphatemia group. Many studies have shown that even a slight increase in serum Pi within the normal range was associated with a higher risk of adverse outcomes significantly in the non-critically ill population. However, this relationship in patients with sepsis has never been studied. In our study, the serum Pi was evaluated as a continuous variable. It showed that minor elevation was independently associated with a higher risk of in-hospital mortality whether in the normal range or not in septic patients.

The mechanism is still unclear. Possible explanations include low muscle strength, subclinical vascular disease, vascular calcification, cardiovascular disorders, etc. (12, 17, 31–34). Several studies have shown that an increase of serum phosphate within the normal range can independently predict a greater likelihood of vascular calcification or increased arterial stiffness in CKD and the general population (32). In Ginsberg et al.’s study, they reported that higher serum phosphate levels, even within the normal range, are associated with microvascular dysfunction in community-living individuals (33). In this study, we found that the higher serum Pi level was associated with a higher norepinephrine infusion rate. This may be attributed to vascular disorders associated with increased serum Pi as described above.

Also, higher quartiles of serum phosphate were found to have a significant association with lower muscle strength and a higher risk of dynapenia (31). This may explain the longer ICU stay associated with hyperphosphatemia in our study.

Whether higher Pi was a direct cause of increased mortality or a marker of disease severity is still unclear. More research studying the potential mechanisms and assessing the potential benefits of lowering serum Pi are needed.

The present investigation had several limitations. First, this study only used serum Pi measurements within the first 24 h of sepsis and did not study the effects of all measurements obtained during ICU stay and the changes in serum Pi on the prognosis of septic patients. Second, the baseline SOFA score was assumed to be zero, as we did not know if the patient has preexisting organ dysfunction before the onset of infection.

## Conclusion

A minor increase of serum Pi, even in the normal range, could be closely associated with a higher risk of in-hospital mortality significantly in septic patients regardless of kidney function, gender, respiratory infection, vasopressor use, and SOFA score.

## Data Availability Statement

The datasets presented in this study can be found in online repositories. The names of the repository/repositories and accession number(s) can be found below: https://physionet.org/content/mimiciv/1.0.

## Author Contributions

ZL, TS, and YH conceived the idea. ZL completed the online training course of data or specimens only research and extracted the date. ZL and TS performed the analysis and drafted the manuscript. YH interpreted the results and helped revise the manuscript. All authors contributed to the article and approved the submitted version.

## Conflict of Interest

The authors declare that the research was conducted in the absence of any commercial or financial relationships that could be construed as a potential conflict of interest.

## Publisher’s Note

All claims expressed in this article are solely those of the authors and do not necessarily represent those of their affiliated organizations, or those of the publisher, the editors and the reviewers. Any product that may be evaluated in this article, or claim that may be made by its manufacturer, is not guaranteed or endorsed by the publisher.
